# Age, Frailty, Resuscitation and Intensive Care: With Reference to COVID-19

**DOI:** 10.3390/geriatrics6020036

**Published:** 2021-04-01

**Authors:** David G Smithard, Nadir Abdelhameed, Thwe Han, Angelo Pieris

**Affiliations:** 1 Department Geriatric Medicine, Lewisham and Greenwich NHS Trust, London SE13 6LH, UK; 2 School of Health Science, University of Greenwich, London SE9 2UG, UK; 3 Geriatric Medicinet, King’s College Hospital, London SE5 9RS, UK; nadir.abdelhameed@nhs.net (N.A.); thwe.han@nhs.net (T.H.); 4 Geriatric Medicine, St Thomas’ Hospital, London SE1 7EH, UK; apieris@nhs.net

**Keywords:** aging, ethics, cardiopulmonary resuscitation, outcome

## Abstract

Discussion regarding cardiopulmonary resuscitation and admission to an intensive care unit is frequently fraught in the context of older age. It is complicated by the fact that the presence of multiple comorbidities and frailty adversely impact on prognosis. Cardiopulmonary resuscitation and mechanical ventilation are not appropriate for all. Who decides and how? This paper discusses the issues, biases, and potential harms involved in decision-making. The basis of decision making requires fairness in the distribution of resources/healthcare (distributive justice), yet much of the printed guidance has taken a utilitarian approach (getting the most from the resource provided). The challenge is to provide a balance between justice for the individual and population justice.

## 1. Introduction

Access to health care is a fundamental human right, but at the same time its availability is finite. Fairness does not mean equal access, as there may be physical, logistical, and technological reasons why equity cannot be guaranteed [1]. Distributive justice provides moral guidance to those making the decisions [2] in the provision of health care. In times when demands on health systems suddenly escalate and/or where health resources are limited (e.g., during the COVID-19 pandemic and the surge in hospital admissions), there are real concerns that the local health system could be overwhelmed by an influx of very unwell people. Where demands are too great or resources so poor that it is not possible [3] to provide the level of medical care to all those that require it (e.g., access to ICU and the requirement for intensive/invasive physiological monitoring including MV), difficult decisions have to be made as to who will be offered or denied access and when any intervention should cease.

As more people survive into adulthood and achieve a greater lifespan, older people are becoming a larger percentage of the world’s population, with 15% of the UK population and 27% of the Japanese population being >65 years [4,5]. Great age is accompanied by many social and health concerns, which results in the accumulation of multiple long-term conditions and increased dependency. As an example, in terms of UK demography, the 85+ age group (very old) is the fastest growing cohort and is set to double to 3.2 million by mid-2041 and treble by 2066 (5.1 million, 7% of the UK population) [4,5,6]. More concerning is that over the last 15 years, there has been an 85% increase in the number of centenarians, and by 2030, this is predicted to reach 21,000 (UK Data) [6]. Chronological aging is not the same as biological ageing. Biological aging is a dynamic biological/physiological process that is influenced by many external factors (environmental, psychological, behavioral, and social). All biological systems are affected by the aging process, but the rate of aging is not uniform across the whole population [7]. With increasing age, there is a gradual decline in physiological reserve, and where this is pronounced and homeostatic mechanisms start failing, frailty is said to occur (24% in those >85 years) [8]. Whilst there is no clear universal consensus regarding the definition of frailty, most researchers and clinicians accept that a loss of biological reserve, immune function [9], a failure of homeostatic mechanisms, a vulnerability to adverse outcomes [10], or a state of increased vulnerability to a poor resolution of homeostasis following a minor stress (e.g., medication change, infection) [11,12,13] are indicators of frailty. 

Admission to ICU is reserved for people who require support for more than one organ system. Patients are admitted to ICU for three main clinical indications: Medical (cardiovascular, respiratory, or gastrointestinal), surgical (cardiac, GI, cancer), where there is a reasonable expectation of life outside of a medical facility [14] and to reduce avoidable mortality [15]. Sepsis is a small percentage of admissions (3.5%) [16]. The demographics will vary depending on whether the ICU provides Level 2 and 3 or just Level 3 care. Access to ICU should be open to all depending on need and in the delivery of justice, but this is not necessarily what happens [16]. One quarter of people admitted to ICU in the USA and Europe are very old (>80 years of age) [17]. With medical innovation and developments, older people admitted to ICU will survive. The literature is confused on the issue of age, with many studies failing to account of comorbidities [18,19,20], resulting in the suggestion that age is the predominant factor of predicting outcome [19]. However, older people are less likely to be admitted to and, if admitted, less likely to be offered intensive intervention (renal support, vasopressors, and MV) [19,20], therefore, it is not surprising that outcomes are poor.

The medical team needs to be able to identify those that are most likely to benefit from admission to ICU following cardiac arrest [21] or organ dysfunction [22]. Secondary analysis of the ICON study has found that those of >80 years of age have twice the hospital mortality following ICU admission than those younger than 50 years, with a rapid increase after 71 years of age [22]. Nobile et al., similarly looking at ICON data, noted that <10% of people leave hospital alive with a good neurological outcome following ICU admission post cardiac arrest [21]. Pietillainen et al., looking at ICU data in Finland, noted that mortality for those of 80 years of age or more was 21.3% in the ICU and 36.2% at 30 days [23]. Other studies have found that age per se is not associated with an increase in mortality, but rather pre-existing frailty [18] and a poor premorbid functional status [15], or whether the admission was planned or unplanned [24].

It is important therefore, that discussions need to be had about the appropriate means of identifying those people, particularly the frail older population, that will benefit from CPR and/or admission to ICU. Whatever decision support tool is designed or employed must be seen to be transparent and fair to all.

## 2. Influence of COVID-19

In May 2020, data (26/05/2020) from NHS England website showed that the total of deaths for COVID patients over 80 years were 13,704 [25]. Data from the Washington DC showed similar results [26]. Department of Health (England and Wales) data show that adults older than 80 years of age accounted for 52% of deaths in the data retrieved on 24/5/2020 [27], but nationally, death secondary to COVID-19, as a proportion of all deaths, is the same for all decades between the ages of 50 and 90+ years [26,27], however, case fatality rates are significantly higher in those over the age of 80 compared to those 40–49 years in all countries [28]. A retrospective case note review of 136 (105 people >60 years) cases undertaken in Wuhan, with laboratory-proven positive COVID -19 and had required CPR whilst an inpatient. Spontaneous return of circulation occurred in only 18 (13.2%), and only 4 (2.9%) survived 30 days. Eleven (10.5%) of those of >60 years of age had a return of spontaneous circulation and, 2 (1.9%) survived until 30 days [29]. These studies confirm that the outcome following CPR and COVID-19 is poor, particularly in older patients [30]. In conclusion, outcome (immediate and 30-day survival) following inpatient CPR is extremely poor in the very old, in the context of COVID-19.

For those critically ill patients with COVID-19 requiring admission to ICU, overall mortality is currently 52% in England and Wales (ICNARC), for those of >70 years of age, mortality is 68.1%, compared to 45.8% between 60 and 69 years, and 23.6% in those of <50 years of age [30]. In the presence of COVID-19, the mortality of older people requiring MV was almost 70% [8] without the presence of any comorbidity, the presence of which would result in a mortality closing in rapidly on 100% [31].

At the height of the COVID-19 pandemic in the UK, the National Institute for Health and Care Excellence (UK) released guidance outlining the importance of identifying frailty, with a Clinical Frailty Score (CFS) = 5 to be the starting point for discussion as to the appropriateness of MV [32]. The purpose is to identify patients who are at an increased risk of poor outcomes and who may not benefit from critical care interventions [30]. Papers looking at the role of frailty and the CFS to predict outcome have come to opposite conclusions [33,34]. It needs to be emphasized that the CFS should not be used as the sole arbiter for admission to ICU and ventilation, or a “get out of jail” card releasing the physician for the obligation to provide treatment [30], but as a pointer to any discussion that takes place.

Would a sensible approach be to look at the presence of frailty using the CFS, combined with the SOFA (Single Organ Failure Assessment) [35] score as part of the ICU assessment? Further research is required to answer this question. Understanding the (f)utility helps to guide decision-making regarding treatment escalation plans, especially in the current environment of COVID-19 pandemic.

## 3. Decision-Making

People make choices every day throughout their life. Many clinical decisions are relatively straightforward, others, particularly those affecting life and death, are more complex. The ethical approach that is frequently adopted is a mixture of distributive justice, egalitarianism, and utilitarianism [36] (Table 1). A balance has to be struck between the needs of the individual and those of the population as a whole [1]. There is, inevitably, a tension between the need to be fair to the individual, ensuring that there is equal treatment for those with the same clinical condition (distributive justice) [37,38], whilst disadvantaging as few people as possible (utilitarian principles). As a result, when resources are reduced or overwhelmed, criteria will change and some people who would otherwise gain access may be denied and or in the case of ICU access, severity will increase [37]. The decision whether to provide a treatment or not is influenced by many competing demands, but the overriding theme must be that any intervention is in the patient’s best interest and provides a net clinical benefit, with minimal harm. Where the evidence of benefit is unknown, a path needs to be navigated such that decisions demonstrate appropriateness, proportionality, and justice. Where this approach is not taken, even the best health care systems may struggle to meet the demands put upon it.

Decision-making requires an evidence base and the experience to utilize the evidence in the best interest of the individual patient, resulting in the best outcome with minimal harm to the individual or others who may ultimately be denied treatment [37,38].

The evidence of benefit achieved from CPR and/or admission to ICU (with or without MV initiation) varies depending on individual patient characteristics [8]. Patients and/or their families may expect everything possible to be done (including admission to ICU), even when the medical team does not think that there is any benefit to be gained and advises against CPR/ICU/MV, because of their wish to maintain life at all costs. Tools to assist the decision-making process are available and should be utilized by the clinician to inform the discussion with all concerned and documentation of the outcome needs to be undertaken in a structured manner [39].

## 4. Decision-Making Tools

Where there is a high demand and limited resources, it is imperative that decision-making is transparent and consistent [40]. The COVID-19 pandemic provided an opportunity to develop pathways and provide guidelines to assist medical staff in decision-making, where CPR and admission to ICU for MV was concerned [40,41,42,43,44]. These documents, although utilizing the evidence available, were largely consensus documents.

Prognostic scoring, using physiological data of people prior to or on admission to ICU, were and is used to triage and describe the population admitted to ICU. The scoring system is used to assist in decision-making and manage costs and resources [45]. The scores were not designed to be used at an individual level [46] but are helpful for standardizing research and comparing the quality of patient care across ICUs [47]. There are no specific algorithms available that accurately identify who should be admitted to ICU and prognostic scoring tools are generally not considered suitable for identifying those likely to have poor outcomes prior to admission to ICU [48]. ICU staff persist in using a plethora of algorithms/decision support tools [45]. Some of these focus on illness severity, others on outcome and a few can be used on a daily basis to follow clinical progress [46].

It is not clear what the best/best combination of tools to predict outcome in older adults is in respect of CPR and ICU admission/MV. Guidance during the first wave of the COVID-19 pandemic in the UK, an algorithm/decision flow chart [32], was produced, which suggested that the Clinical Frailty Score (CFS) [49], a 9-point ordinal (categorical) scale [49], could be used to identify/triage/select patients for ICU admission. This was subsequently amended to ensure that the CFS was not applied to younger adults with fixed neurological conditions (e.g., learning difficulties, cerebral palsy) [50].

The CFS is straightforward to use, and training is not required [51], with evidence to show it is useful in contributing to predict the outcomes of multiple acute medical and surgical scenarios [35,52,53,54,55]. The CFS has not been widely validated in younger populations (below 65 years of age), who have a coexisting long-term condition (e.g., cerebral palsy, head injury, and in those with learning disabilities) and should be used with extreme caution. A recent paper by Hewitt et al. suggests that the CFS can help in predicting the prognosis of those people admitted to hospital, in the context of COVID-19, from the age of 50 years [33], but its validity in the acute scenario has been questioned [34].

At the time of admission to ICU, prognostic assessments are undertaken within the first 24 h, e.g., The Simplified Acute Physiologic Score III (SAPS III) [56], the Single Organ Failure Assessment (SOFA) [35], and the Acute Physiology and Chronic Health Evaluation II (APACHE II) [57] (Table 2).

Dossett et al. found that the APACHE II score was useful for predicting mortality but not perfect [62] in all situations. SAPS III had a predicted hospital mortality of 84% sensitivity and 66% specificity, and APACHE II 69% and 66%, respectively. Hope et al. have shown that the presence of frailty increased hazard ratio (HR) in ICU mortality by 1.27, in those >75 years over and above that expected [63].

None of these scores utilizes a measure of frailty (though the presence of comorbidities and long-term conditions is a pointer towards frailty). The SOFA score has been proposed as a tool to be used in the triage process, determining access to mechanical ventilation on critical care units in the event of an influenza pandemic where the number of patients that may need ventilation exceeds the number of patients that can be ventilated [35,64]. Proponents advocating the use of SOFA have cited the relative accessibility and objectivity, i.e., due to the use of six measured organ dysfunction-related parameters in calculating the score. It is accessible as the six parameters are already measured routinely as part of critical care medicine and nursing and can be calculated during or prior to moving onto a critical care unit. The score can be calculated by inputting the six measured results into freely available online tools.

Any scores need to be well validated, calibrated, and discriminative. Unfortunately, none of these are beneficial in extremes of illness [46] or validated in extremes of old age [17]. Prognostic ICU scores, like many scales in hospitals, are designed for a younger, fitter population [17] and cannot be considered sufficiently credible or valid to be applied to older people [65]. Preadmission frailty and performance score would be a better predictor of outcome.

## 5. Benefit of CPR?

Firstly, is it likely to be successful and is there a good chance of the patient leaving the hospital alive, and secondly, will there be a bed in ICU and a mechanical ventilator available if required [66,67]. Ethically, it is inappropriate to offer a treatment to someone if that treatment has little or no utility [68]. Therefore, there needs to be an understanding of the likely effectiveness of CPR in the old and very old population [69].

A large observational study of 11,396 patients in the Swedish Cardiopulmonary Resuscitation registry between the years of 2007 to 2015 [70] documented that the 30-day survival rate decreased with advancing age (14% for those of ≥90 years of age, 20% for those of 80–89 years of age, and 28% for those of 70–79 years of age) [16]. Similarly, a systematic review of 29 articles (Dutch and English) found that survival to discharge following inpatient CPR was 18.7% for 70–79, 15.4% for 80–89, and 11.6% for 90 and above [70]. People undergoing CPR due to cardiac arrhythmia (ventricular tachycardia and fibrillation) had a better outcome (≥90 had a 40.9% survival from ventricular arrhythmias and only 3.3% otherwise) [65]. The conclusion must be that CPR should be targeted and not offered to all at the extremes of age.

## 6. Benefit of Mechanical Ventilation

Admission to ICU and the provision of MV may be the correct place to manage the physiological complications in critically ill patients. Admission to ICU and MV are not without harm and may result in prolonged medical complications and difficult discussions around (dis)continuation of management [8]. Research has shown that the presence of comorbidities and age correlates closely with worsening outcomes if MV is provided [71]. Haq et al. [71] in a retrospective study of people of >90 years of age admitted post-surgery to ICU, found the overall mortality was 15.7% (14/89). Vosylius et al. performed a prospective observational study in Lithuania, (477 patients > 75 years (75–84, 85+)) and noted that mortality doubled for those >75 years compared to <65 years (39% vs. 19% *p* < 0.001) [72]. In a retrospective analysis increasing age, particularly in the presence of cerebrovascular and cardiac disease [52,53], was associated with increased mortality and length of MV. MV for 7–10 days, only 5% of those ≥85 years survived to be discharged home [54]. Frengley et al. (2014) reported that increasing age was associated with a decreased ability to be weaned off the ventilator, but most of this was associated with increasing comorbidity rather than age alone [55]. The decision to offer MV to people of extreme age must only be undertaken after careful assessment as the benefit is small and the potential for harm is always present [36]. Mortality in ICU is often secondary to organ failure and sepsis [73]. The presence of sepsis was associated with a doubling of mortality in those >80 years compared to <50 years [22]. Increasing frailty and comorbidity burden/organ dysfunction results in a higher rate of MV withdrawal, and mortality [17,21]. Age alone is not a good predictor of outcome for anyone being admitted to ICU, the presence of frailty, poor performance status, or multiple comorbidities are [17]. Pietillainen et al., looking at ICU data in Finland, noted that mortality for those of 80 years of age or more was 21.3% in the ICU and 36.2% at 30 days. Mortality was greater in those with a poor premorbid functional status [23].

## 7. Ethical Concerns

Everyone is unique, and as such anyone and everyone, irrespective of age or medical illness, is entitled to be provided with the medical care that is appropriate to their needs [8,40]. What is appropriate and possible and what people expect (or demand) may be two different things and these may be poles apart. When clinical decisions are being made, it must be remembered that all people have intrinsic value, an intrinsic dignity, which is absolute and not relative to others, and as such, life should be protected and respected in accordance with the fundamental requirement of justice [24]. This holds true irrespective of the times we live in. It is as true today as much as it has been in the past and should be going forward.

When the first wave of the COVID-19 pandemic was at its peak hospital admissions, statements issued by various medical organizations were broadly aligned and stated that [38,40,41,72] equal concern and respect is the fundamental approach’. The BMA [42] states that there should be ‘equitable concern for all’, and the Royal College of Physicians [43] comment that ‘fairness is the best way to understand the ethical problem that clinicians are likely to encounter [74]. However, these do not help when demand outstrips supply, and decision-making has to be rapid, will medical staff adopt a blanket or fixed approach to the process such that older people would be denied care based on the single criterion of age alone. However as people approach a great age, cardiac, cognitive/neurological, and respiratory comorbidities are often present, all of which contribute to morbidity and prognosis from an acute (fulminating) illness.

Therefore, how should we decide on who should be resuscitated and who should be offered mechanical ventilation? These decisions are being made daily, but in the time of crises, they seem more brutal and exposed. The basis of any treatment offered should be, following the principles of distributive justice and utilitarian principles, that access to health care should be on the basis of need and the person being offered or undergoing the treatment is going to gain an overall benefit. In other words, the benefit to be accrued from the treatment is greater than any harm that may occur and that no, or limited, harm will come to others [1,37,38,74,75,76,77].

Before implementing any management plan, especially those that are invasive or heavily burdensome or may carry a significant risk [15,75], the purpose of that plan needs to be understood by all concerned. It is imperative that medical staff and family members do not employ or demand interventions that are harmful with no obvious benefit being accrued or insist on prolonging death where life can never be sustained [78,79].

Is the treatment being offered part of normal care? Providing antibiotics, fluids, and nutrition is normal care. Failure to do so may result in the death of the patient, even if that person is not dying, and not at the end of life. A contentious point in the UK is that the General Medical Council suggests that anyone is at the end of life at any time during their predicted last year of life [76]. However, CPR and MV are extraordinary treatments and therefore, would not necessarily need to be offered to all [74,75]. The final decision on the provision of care resides with medical staff and, in the case of CPR, this is qualified by the clinical facts and the provision of appropriate aftercare, which may include the availability of an ICU bed [67].

So what should be done when resources are so stretched that options are limited? Who gets the offer of treatment? Should/can someone be taken off MV, if someone else is deemed to be more worthwhile, if they are not improving fast enough [15,75,76,80]? Inevitably, this suggests that there will be some inequality in provision which has the potential to be dependent on who is making the decision, who you are, and where you live. Who should decide who is to be offered MV, the latest expensive drug, or expensive complex surgery? There is a role for an institutional ethics committee to be the final arbiter of such decisions.

The withholding of appropriate treatment without good reason is as ethically and morally inappropriate as offering treatment that is known to have no benefit. Decisions must be made at a patient level and no one should be denied treatment just on the basis of who they are [24,75]. Individual decisions cannot be made in a moral vacuum and inevitably, any choices affecting one person will inevitably affect others and some may inevitably come to harm, particularly when resources are limited [79,81]. Decision-making should be a collective approach, using the “multiple eyes principle” [76] and not leave one person exposed to any recrimination at a later date.

There remains a tension between the rights/expectations of the individual and those of wider society [80]. Health care provision will need to find a balance between equity, equality (distributive justice), or utility of care [61]. In times of “stress”, a utilitarian approach is adopted, in that there should be an effort to make sure that there is the greatest return on the investment, or maximizing the utility of any intervention [76,81]. In healthcare terms, outcomes expected could be more people surviving, more productive years gained [77,81] but the downside is others will be denied care. Any guidance/policy written to ensure transparency has an inherent danger that it will differentially and (in)directly discriminate against older people, those with long-term conditions, and those with poor access to health care.

The commencement of any acute medical intervention must have a plan as to when and why its utility should be stopped. It is justified to stop or withdraw a treatment if it no longer serves its purpose and has become futile or excessively burdensome [15,75].

Decision-making is not an exact science it is an art. Decisions must be informed by the available clinical facts and scientific evidence. At critical time points, judgment calls/decisions may appear to be cut and dried and made without compassion to other members of the health care team.

Those undertaking the action as a result of the decision may not fully understand the reasoning or facts of the decision despite the decision being right, they may believe it to be wrong and therefore, suffer moral harm (Table 3). The exact opposite is where a purposively bad or wrong decision is made, which is understood to be wrong, but coercion or force is employed to ensure the action is taken, resulting in moral injury.

Health care professionals are often wary concerning guidelines and their legal implications. Guidelines provide a framework to direct/inform decision-making, to ensure some consistency in implementation and clarity/transparency to the process, ensuring that justice and fairness are delivered, therefore, providing clinicians with some legal protection. Since the publication of the BMA guidelines, there have been papers questioning the legality of the advice. Lidell et al. [82] summarize the information and conclude that any decisions need to be aware of the violation of legal rights with contravention of the Human Rights Act [83], Mental Capacity Act (2005) [84] and the National Health Service Act [85], that may occur, in particular if MV is removed from a person who is not deteriorating [73]. The Government has sought to provide mitigation by way of the Corona Virus Act 2020 [80] to offer some protection to health care staff.

## 8. Conclusions

All people have intrinsic dignity, and as such, life is to be valued highly. Dignity involves showing respect to others, irrespective of social status. Treat them justly and with mercy and compassion (Misericordia), as we would like ourselves to be treated. The discussion around cardiopulmonary resuscitation and admission to ICU and MV needs to be had and appropriate decisions made [38]. The admission of very old frail adults with poor functional status being exposed to CPR and ICU may be inappropriate in any clinical situation, let alone in the presence of COVID-19. In clinical situations, difficult decisions need to be taken frequently. COVID-19 has brought decision-making into sharp relief and has raised the question as never before, whether age, age and comorbidities, or premorbid functional status/frailty are the best indicators of outcomes following CPR and admission to ICU. The literature suggests that premorbid functional status or frailty [17,18] are the best indicators, particularly when the admission is unplanned [24].

Despite best intentions, it will not be possible to please everyone. To retain confidence in the health care team, the decision-making process needs to be fair, transparent, and to demonstrate consistency, and above all, show compassion.

## Figures and Tables

**Table 1:** Comparison of distributive justice, egalitarianism, and utilitarianism.

Distributive Justice	Egalitarianism	Utilitarianism
Fairness in resource allocation	Treatment according to need	Best result for all

**Table 2:** Components of Prognostic Scores.

	Diagnosis	Physiology (Clinical and Lab)	Age	Comorbidities	Glasgow Coma Score	Timing (of Admission—h)	Reference
SAPS III	X	X	X	X	X	1	[56]
APACHE		X	X	X		24	[57]
SOFA		X			X	34	[35]
MODS		X				24	[58]
LODS		X			X	24	[59]
MPM II	X	X	X	X		24	[60]
ODIN		x			x	24	[61]

SAPS III (Simplified Acute Physiologic Score), APACHE (Acute physiology and chronic health evaluation), SOFA (Single Organ Failure Assessment), MODS (Multiple organ dysfunction score), LODS (Logistic organ dysfunction score), MPM II (Mortality probability models), ODIN (Organ dysfunctions and/or infection). X: component is part of score.

**Table 3:** Moral effects of decision-making.

Moral	Decision Made	Understanding of the Facts	Subsequent Understanding of the Decision
Distress	Right	Right	Wrong (action justified but subsequently found to be wrong)
Burden	Right	Right	Right (action justified and remains so)
Harm	Right	Wrong	Right (action justified and remains so, error is in the understanding)
Injury	Wrong	Wrong	Wrong (action not justified and perhaps immoral or even evil)
